# Pucker sign in irreducible posterolateral knee dislocation

**DOI:** 10.11604/pamj.2018.30.153.15365

**Published:** 2018-06-20

**Authors:** Sanjiv Fitz-Morris Gray, Beatrice Esther Dieudonne

**Affiliations:** 1 University of Central Florida College of Medicine Orlando, Florida, USA

**Keywords:** Knee dislocation, posterolateral dislocation, USA

## Image in medicine

24 years old male seen in the trauma bay involved in a motor vehicle accident with rollover landing on its roof. He was partially ejected with his right leg hyperflexed and entrapped. The patient was stable with complaints of right knee pain. There was dimpling of the right distal medial thigh (A, B). After unsuccessful attempts at closed reduction, the patient was taken for open reduction. There was entrapment of the femoral condyle in the extensor mechanism. The soft tissues were released and the condyle reduced back into its normal position. The extensor mechanism was then repaired. The patient had palpable posterior tibial and dorsalis pedis pulses after completion of the procedure. The mechanism for posterolateral knee dislocation is valgus and rotational stress during knee flexion. The medial cruciate ligament, anterior cruciate ligament, and the posterior cruciate ligament are usually injured in posterolateral knee dislocation with incarceration of the capsule and ligamentous structures in the intercondylar notch. Early open reduction is indicated as it is generally considered irreducible by closed reduction. The "pucker sign" is considered pathognomonic. On Radiographs with attention to the patella shows the patella maintaining its in-line attachments to the tibial tuberosity and is reduced with respect to the proximal tibia but dislocated with respect to the distal femur. At the follow up the patient is ambulatory after rehabilitation.

**Figure 1:**
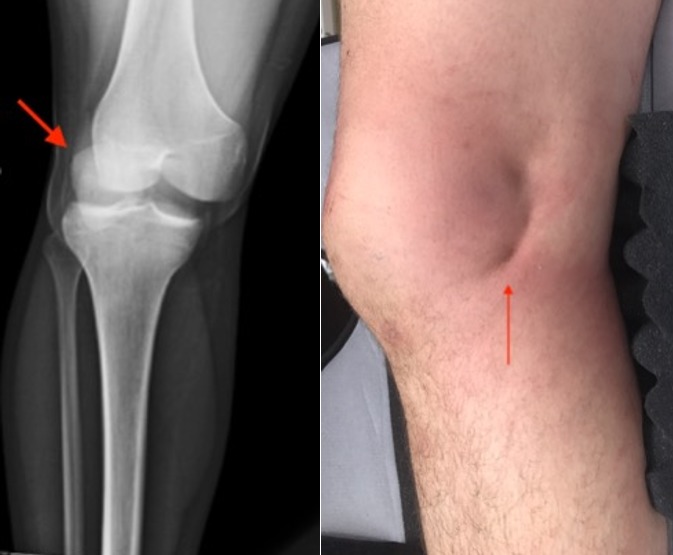
Xray showing posterolateral dislocation of the knee. Note the maintained alignment of the patella and the tibia with respect to the femur; pucker sign noted medially

